# Emerging Therapeutic Modalities and Pharmacotherapies in Neuropathic Pain Management: A Systematic Review and Meta-Analysis of Parallel Randomized Controlled Trials

**DOI:** 10.1155/prm/6782574

**Published:** 2024-12-26

**Authors:** Ernest Kissi Kontor, Catherine Wellan, Hafiz Mohammad Maaz, Daha Garba Muhammad, Almonzer Al-Qiami, Amin Sharifan, Jessica Kumah, Hester Lacey, Abdelmonem Siddiq, Nityanand Jain

**Affiliations:** ^1^Department of Theoretical and Applied Biology, Kwame Nkrumah University of Science and Technology, Kumasi, Ghana; ^2^Clinical Laboratory, Evergreen Health, Monroe, Washington, USA; ^3^Faculty of Medicine, Quaid-e-Azam Medical College, Bahawalpur, Pakistan; ^4^Department of Physiotherapy, Bauchi State Ministry of Health, Bauchi, Nigeria; ^5^Faculty of Medicine, Kassala University, P.O. Box 1115, Kassala, Sudan; ^6^Department for Evidence-Based Medicine and Evaluation, Universität für Weiterbildung Krems, Dr. Karl Dorrekstrasse 30, Krems 3500, Austria; ^7^Department of Occupational and Environmental Public Health, University of Toronto, Toronto, Canada; ^8^Faculty of Medicine, Brighton and Sussex Medical School, University of Sussex, 94 N-S Rd, Falmer, Brighton BN1 9PX, UK; ^9^Faculty of Pharmacy, Mansoura University, Mansoura 35516, Egypt; ^10^Statistics Unit, Riga Stradinš University, 16 Dzirciema Street, Riga LV-1007, Latvia

**Keywords:** capsaicin, EMA401, neuropathic pain, Sativex, spinal cord stimulation, transcranial magnetic stimulation, transcutaneous electrical nerve stimulation

## Abstract

**Background:** Neuropathic pain (NP) is a chronic condition caused by abnormal neuronal excitability in the nervous system. Current treatments for NP are often ineffective or poorly tolerated. Hence, we reviewed the efficacy and safety of novel drugs or devices that target neuronal excitability in NP patients compared with placebo, sham, or usual care interventions.

**Methods:** Six databases were searched for parallel randomized controlled trials (RCTs) reporting novel devices (rTMS, SCS, and TENS) or drugs (EMA401, capsaicin 8% patch, and Sativex) for NP. Data were extracted and quality was assessed using the ROB2 tool. The random-effects inverse variance method was used for analysis.

**Results:** In our review of 30 RCTs with 4251 participants, device-based interventions were found to be more effective in reducing pain scores than control interventions (SMD = −1.27, 95% CI: −1.92 to −0.62). However, high heterogeneity was seen (*p* < 0.01, *I*^2^ = 91%), attributable to the etiology of NP (*R*^2^ = 58.84%) and year of publication (*R*^2^ = 49.49%). Funding source and type of control comparator were ruled out as cause of heterogeneity. Although drug interventions did not differ from placebo interventions in absolute pain reduction (SMD = −1.21, 95% CI: −3.55 to 1.13), when comparing relative change in pain intensity from baseline, drug interventions were found to be effective (SMD = 0.29, 95% CI: 0.04–0.55). Asymmetry in the funnel plot was visualized, suggesting publication bias. Certainty of evidence was very low according to GRADE assessment.

**Conclusions:** Our review indicates that device-based interventions are more effective than control interventions in reducing pain intensity in NP. Nevertheless, available evidence is limited due to heterogeneity and publication bias, prompting the need for more high-quality RCTs to confirm the efficacy and safety of these interventions.

## 1. Introduction

Neuropathic pain (NP) is a chronic and debilitating condition that affects the quality of life of millions of people worldwide [1, 2]. It arises from damage or lesions of the somatosensory system [3], which results in abnormal processing of sensory information and increased neuronal excitability in the peripheral and central nervous system (PNS and CNS). NP has various etiologies and manifestations, depending on the anatomic localization and the underlying pathological mechanisms. Some of the common causes of NP are metabolic disorders, viral infections, autoimmune diseases, chemotherapy, trauma, inflammation, hereditary neuropathies, and channelopathies [4]. Despite the availability of several pharmacological and nonpharmacological interventions for NPs, most of them have limited efficacy and tolerability [5], and there is a high unmet need for novel and effective treatments.

Recent advances in the understanding of the neurobiology of NP have led to the development of new therapeutic approaches that target neuronal excitability or pain intensity, which is a key factor in the generation and maintenance of NP. Literature reviews have described approaches such as sodium channel blockers, potassium channel openers, calcium channel modulators, and neuromodulation devices, among others [6, 7]. These interventions aim to restore the normal balance of neuronal activity and reduce the aberrant firing of pain-related neurons in the PNS and CNS. However, the effects of these novel treatments on neuronal excitability in NP are not well characterized and require systematic review and synthesis.

A literature review by Bernatoniene et al. explored novel pharmacotherapies and devices targeting neuronal excitability in NP [7]. However, to date and to the best of our knowledge, a meta-analysis evaluating the efficacy of these novel treatments has not been reported. Hence, this meta-analysis aims to provide a comprehensive and up-to-date synthesis of the neurobiological effects of emerging treatment candidates targeting neuronal excitability in NP. Additionally, this analysis will critically assess the existing literature and highlight areas for future research.

## 2. Methods

Before protocol preparation, we searched the recent scientific literature, Drugs.com, and the USA Food and Drug Administration (FDA) database for treatments that have been approved or are under investigation for NP in the last five years and that have paucity of literature in terms of synthesized meta-analytical evidence [5–11]. This led us to identify six emerging drugs and devices, namely, Qutenza (capsaicin 8% patch), Sativex, EMA401, repetitive transcranial magnetic stimulation (rTMS), spinal cord stimulation (SCS), and transcutaneous electrical nerve stimulation (TENS).

The findings of this systematic review were reported according to the Preferred Reporting Items for Systematic Reviews and Meta-Analyses (PRISMA) 2020 guidelines [12]. The review protocol was prospectively registered on PROSPERO (CRD42023465305) on October 1, 2023. Deviations from the registered protocol are reported in Supporting File 1.

### 2.1. Study Objectives

To systematically formulate the search strategy, the patient, intervention, control, outcome, and study design (PICOS) scheme was used (Table 1). The PICOS scheme was based on the clinical question to determine the effects of emerging therapies targeting neuronal excitability on pain intensity or change from baseline (primary outcome) and adverse event measures (secondary outcome) in patients with NP compared to placebo, sham, or standard care. NP was defined as pain caused by damage or dysfunction of the somatosensory system, diagnosed by a validated tool or established clinical criteria. Secondary outcomes included quality of life outcomes such as dizziness, headache, hypertension, nausea, diarrhea, and vomiting.

### 2.2. Search Strategy and Sources

Six electronic databases (PubMed, MEDLINE via Ovid, Scopus, EBSCO-CINAHL, Embase via Ovid, and Web of Science Core Collection) were searched independently from inception to June 2024 by two authors (E.K.K. and J.K.). A combination of keywords derived from the PICOS scheme and MeSH terms related to NP and the specific interventions of interest was used to identify potential studies. The search was restricted to studies published in English and the retrieved records were imported into the Covidence software, automatically removing duplicates. The full search syntax for each database is provided in Supporting File 1.

### 2.3. Inclusion and Exclusion Criteria

In this review, we included only parallel RCTs. The trials were included if they compared the effects of an emerging drug or device targeting neuronal excitability with a placebo, sham, usual care, or another standard active intervention on NP of any etiology in adults ≥ 18 years. We excluded quasi-experimental studies, observational studies, cross-over RCTs, noncomparative studies, case reports, case series, reviews, commentaries, and letters as well as studies that involved children or adolescents (< 18 years), animals, or in vitro models.

Letters were considered if they reported preliminary findings of the trials and full published trials were not available. However, if full trial data were available, then letters were disregarded. Additionally, studies that used nonvalidated scales or tools and trials in which active drugs used were the same as the intervention but with different concentrations, models, frequencies, or doses as a comparator were excluded. Non-English trials were also excluded.

### 2.4. Screening and Data Collection

The titles and abstracts of the retrieved records were screened for eligibility by five reviewers (E.K.K., C.W., D.G.M., H.M.M., and J.K.) using the Covidence software. Each study was evaluated independently by at least two reviewers. All reviewers were blinded to the evaluation of the other reviewers to minimize selection bias. Full texts of the potentially eligible records were obtained and assessed for inclusion by the same reviewers. Any disagreements were resolved by discussion between the reviewers who reviewed the study and an independent third author not involved in the screening process (N.J.). 95% agreement was recorded in the study screening phase.

### 2.5. Data Extraction

The data extraction process resembled the screening process with the same five reviewers independently extracting data using a standardized form. Data from each study were extracted independently by at least two reviewers and cross-checked by a third author not involved in the extraction process (N.J.). 100% agreement was recorded in data extraction. The following parameters were extracted from the included studies—study characteristics (such as authors, year, country, design, sample size, duration, and funding source), population characteristics (such as age, sex, diagnosis, and duration of NP), intervention and comparator details (such as type, dose, frequency, duration, and mode of administration), outcome measures (such as type, time points, and units), and results (such as means and standard deviations). Data extraction sheet is provided in Supporting Files 2 and 3.

### 2.6. Quality Appraisal and Certainty of Evidence

The risk of bias in the included studies was independently and blindly assessed by two authors (D.G.M. and H.M.M.) using the Cochrane Risk of Bias tool for RCTs (ROB2) [13]. Any discrepancies in risk of bias assessment were resolved by discussion and input from other authors not involved in the quality appraisal phase. The GRADE (Grading of Recommendations Assessment, Development, and Evaluation) approach was used to assess the strength and certainty of summarized evidence for primary outcomes of interest [14].

### 2.7. Data Synthesis and Analysis

A pairwise meta-analysis was performed for the studies that reported the same intervention, comparator, and outcome using the <*meta*> package in R studio v4.3.2. Data were pooled using the Mantel–Haenszel random effect model and forest plots were created to visually analyze the results. Standardized mean difference (SMD) with 95% confidence intervals (CIs) was calculated for continuous outcomes using Hedges' g method, while risk ratio (RR) with 95% CIs was used for dichotomous outcomes. SMD was used because it allows for the comparison of effect sizes across studies that use different scales to measure the same outcome, providing a standardized measure of effect. A *p* value of < 0.05 was considered statistically significant.

When results were reported in other statistical summaries than mean and standard deviation, they were converted to mean and standard deviation using the formulas reported elsewhere [15]. When needed, data were obtained from graphs using a web-based validated tool (WebPlotDigitizer; Automeris) [16]. The *<robvis>* package in R (version 4.3.2) was used to create the risk-of-bias chart and refined in Microsoft PowerPoint. For the assessment of study heterogeneity, we used the *χ*^2^ test and the Higgins-*I*-squared (*I*^2^) model for subgroup differences. Subgroup analyses were conducted based on the type of intervention and type of drug or device to explore potential sources of heterogeneity (if any). Meta-regression was performed to examine the relationship between the effect size and possible covariates.

Publication bias (if *N* ≥ 10) was evaluated using Egger's test and contour-enhanced funnel plots at *p* < 0.01, 0.01 < *p* < 0.05, 0.05 < *p* < 0.10, and *p* > 0.10. The trim-and-fill method adjusted for funnel plot asymmetry using L-estimator, while the restricted maximum-likelihood estimator was used for tau squared (*τ*^2^). *Q*-Profile method provided the confidence interval of *τ*^2^ and *τ*. Limit meta-analysis was based on the expectation (beta0) adjustment method. The <*metasens*> package was used for the limit meta-analysis. Code used for the present analyses is provided in Supporting File 4. The PRISMA checklist is provided in Supporting File 5.

## 3. Results

**Figure 1:**
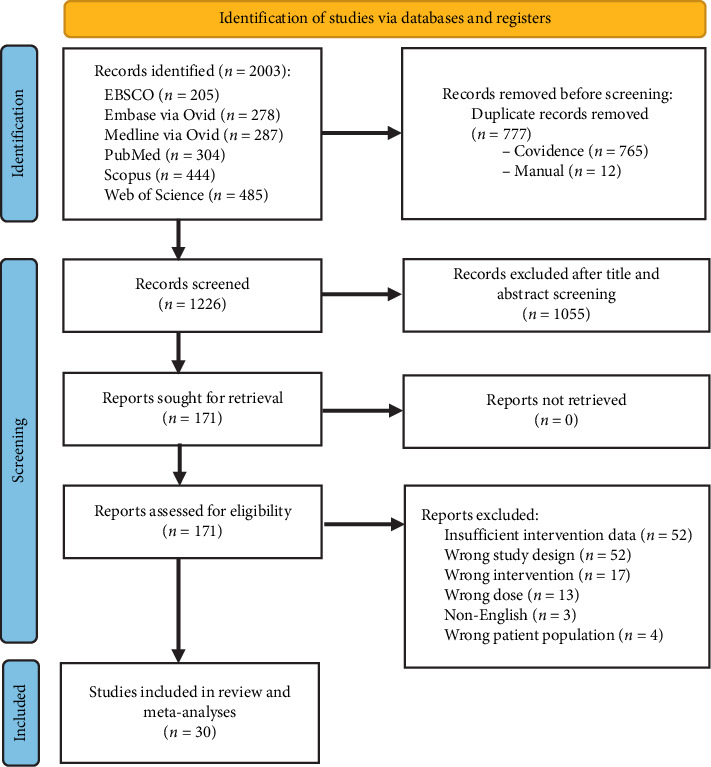
PRISMA flowchart for the present review.

We identified 2003 records from the database search, of which 171 were eligible for full-text screening after removing duplicates and applying title and abstract screening criteria. We further excluded 141 studies that did not meet our inclusion and exclusion criteria, resulting in 30 studies with 4251 participants for the meta-analysis (Figure 1) [17–46]. For the purposes of the present review and meta-analyses, the 30 included publications reported results from 33 different trials—one paper by Rice et al. [39] reported on three separate trials (EMPHENE-25 mg and 100 mg and EMPADINE trials). Similarly, the paper by Pei et al. [43] reported two trials with different rTMS frequencies (10 Hz and 5 Hz).

Nonetheless, we did not consider the open-label extension study reported by Backonja et al. [33] in the present study since the same participants from the double-blind, parallel assignment RCT were rolled-over to the extension study. Among the trials, 13 trials were multicentered with 10 of them being international trials conducted across multiple countries. The earliest trials were published in 2005 with data collection and patient recruitment beginning as early as 2001-2002. 14 trials were funded by private companies, six trials were funded by national and regional government agencies, three trials were funded by the universities, and four trials did not report funding details while two trials were self-supported by the authors. The rest of the trials had multiple sources of funding. Trial characteristics are presented in Table 2.

### 3.1. Risk of Bias Assessment

**Figure 2:**
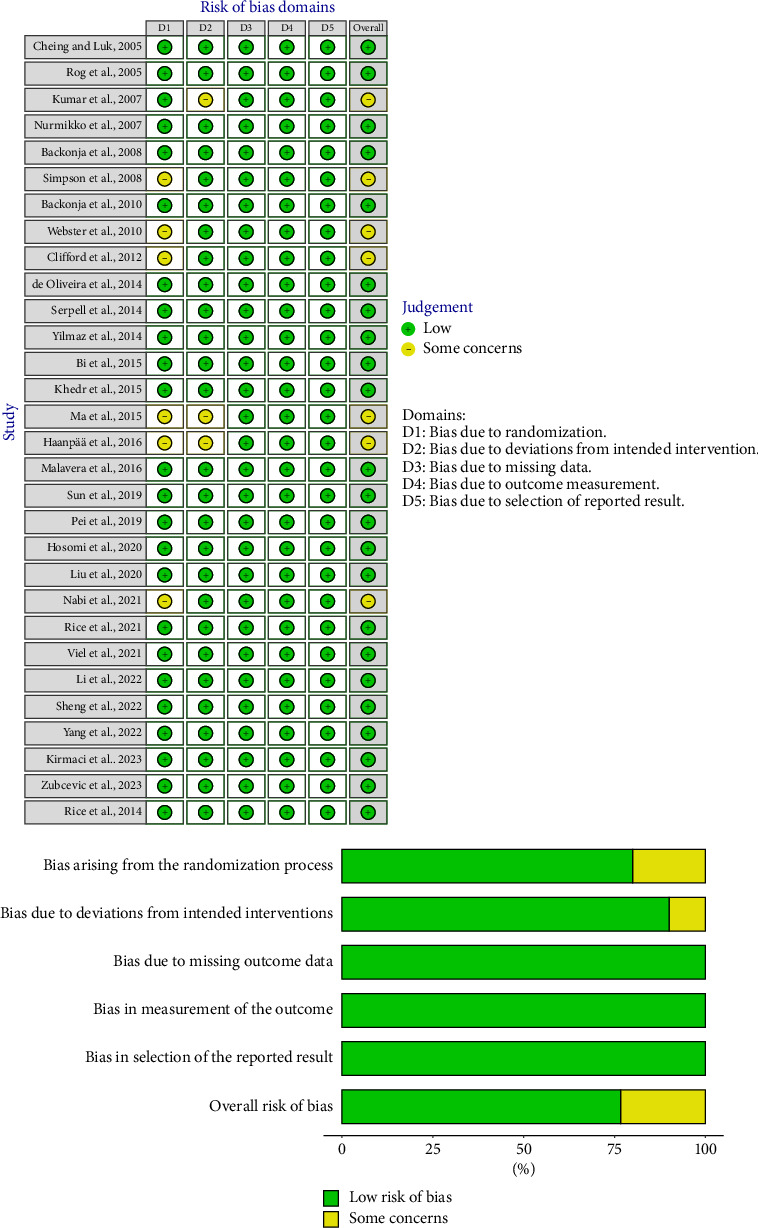
Risk of bias quality appraisal of the included publications according to the Cochrane ROB2 tool.

We assessed the risk of bias for the included studies using the Cochrane risk of bias ROB2 tool (Figure 2). Most of the studies (76.7%) had a low risk of bias across all domains, while seven studies (23.3%) had some concerns, mainly due to unclear or inadequate reporting of the randomization process. None of the studies had a high risk of bias. Accordingly, all studies were considered appropriate for inclusion in the present review.

### 3.2. Meta-Analysis of Primary Outcomes

#### 3.2.1. Pain Intensity

**Figure 3:**
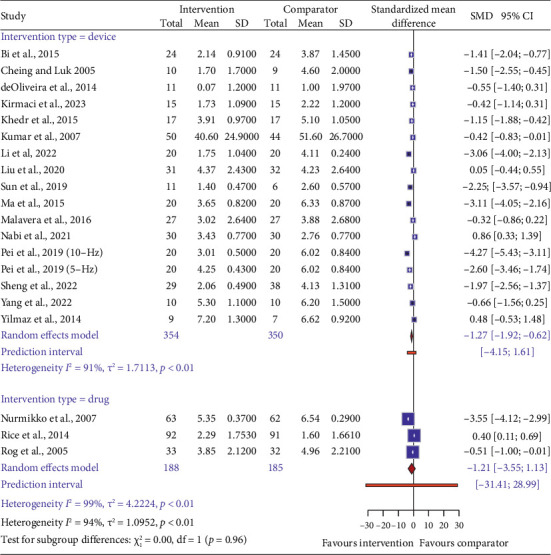
Comparison of the effectiveness of device and drug interventions for neuropathic pain (primary endpoint). The figure displays the SMD and 95% confidence intervals (CIs) of each study comparing an intervention with a comparator for reducing neuropathic pain. The zero line represents no effect of the intervention. The size of the blue squares reflects the study weight in the meta-analysis. The red diamonds show the pooled SMD and 95% CI for each intervention type. The red lines indicate the prediction intervals, which estimate the expected SMD and 95% CI for a new study within each intervention type.

We performed a meta-analysis of 20 studies that reported pain intensity as an endpoint outcome measure. Among them, 17 trials reported on device interventions while the remaining three trials reported on drug interventions (Figure 3). The pooled estimate showed that the participants who underwent device interventions had lower mean pain scores than the comparator group (SMD = −1.27, 95% CI: −1.92 to −0.62). However, significant statistical heterogeneity was observed between the trials (*p* < 0.01, *I*^2^ = 91%). The predictive interval, which reflects the expected range of effects in similar future studies, ranged from −4.15 to 1.61, indicating a high degree of uncertainty and heterogeneity.

Regarding the trials with drug interventions, no difference in mean pain scores was observed between the experimental and comparator groups (SMD = −1.21, 95% CI: −3.55 to 1.13). Statistical heterogeneity remained significantly large (*p* < 0.01, *I*^2^ = 99%) with a large prediction interval (−31.41 to 28.99).

**Figure 4:**
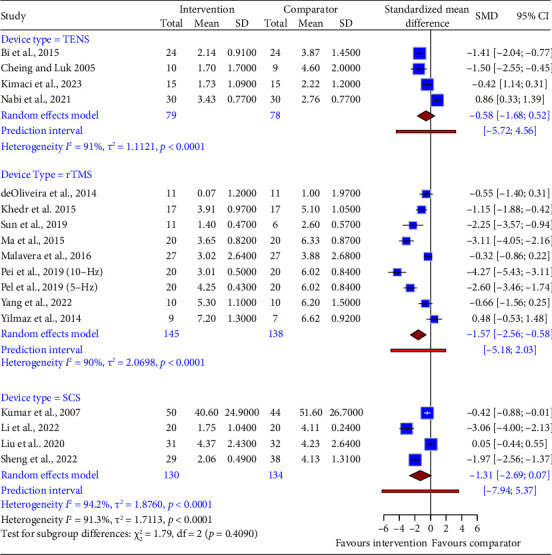
Subgroup comparison of the effectiveness of different device types for neuropathic pain (primary endpoint). The figure displays the SMD and 95% confidence intervals (CIs) of each study that evaluated a device type (TENS, rTMS, and SCS) against a comparator for reducing neuropathic pain. The zero line represents no effect of the device type. The size of the blue squares reflects the study weight in the meta-analysis. The red diamonds show the pooled SMD and 95% CI for each device subtype. The red lines indicate the prediction intervals, which estimate the expected SMD and 95% CI for a new study within each device subtype.

Next, we conducted a subgroup analysis of the device interventions to identify the source of heterogeneity. We found that only interventions with rTMS had an effect on NP pain intensity reduction compared to the comparator group (SMD = −1.57, 95% CI: −2.56 to −0.58; Figure 4), with a predictive interval ranging from −5.18 to 2.03. There was substantial heterogeneity noted within and between the device subgroups.

**Figure 5:**
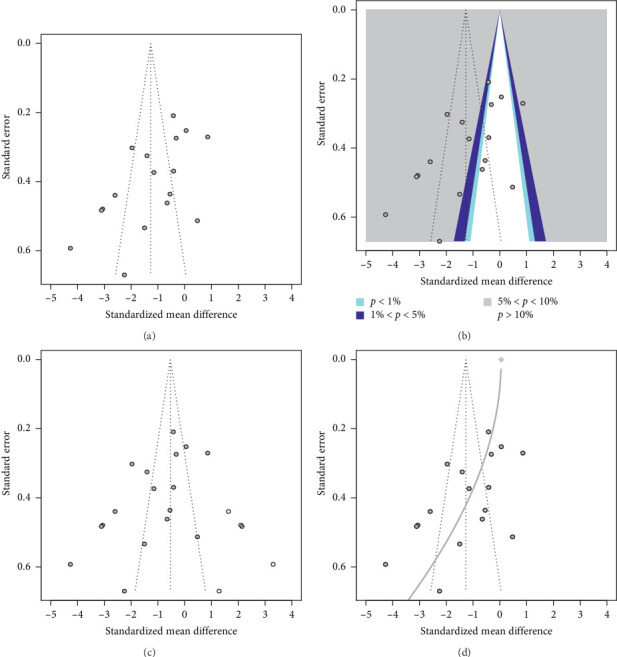
Contour-enhanced funnel plots assessing publication bias in device intervention studies. Each round dot signifies an individual trial. (a) Standard funnel plot showing publication bias in 17 observations. (b) Contour-enhanced funnel plot for the 17 observations. (c) Trim-and-fill funnel plot illustrating bias after adding five hypothetical studies (white dots) to the original 17 observations (gray dots). (d) Limit meta-analysis funnel plot demonstrating increasing bias due to small-study effects as the standard error rises. The gray diamonds mark the adjusted average effect size when the standard error on the *y*-axis is zero.

We also evaluated the presence of publication bias using a contour-enhanced funnel plot (Figure 5) and Egger's test. The funnel plot showed an asymmetrical distribution of the studies, with a lack of small studies with large effect sizes favoring the comparator group. This suggested the possibility of publication bias or other small-study effects. Egger's test confirmed the presence of significant publication bias (*p* < 0.01). Among the 17 publications, 10 were statistically significant at 0.05 < *p* < 0.10, one at 0.01 < *p* < 0.05, and six were not significant.

The trim-and-fill method was applied to assess potential publication bias. A total of 22 studies were included, with five imputed to adjust for funnel plot asymmetry. This resulted in 881 observations, with 445 in the intervention (device) group and 436 in the comparator group. The random effects model yielded an SMD of −0.54 with a 95% CI ranging from −1.33 to 0.26 (*z* = −1.33, *p*=0.184), indicating no statistically significant effect. Substantial heterogeneity was observed, with *τ*^2^ at 3.39 (95% CI: 1.94–7.36), *τ* at 1.84 (95% CI: 1.39–2.71), *I*^2^ at 93.7% (95% CI: 91.6%–95.2%), and H at 3.97 (95% CI: 3.45–4.57). The heterogeneity test was significant (*Q* = 331.29, df = 21, *p* < 0.0001), confirming considerable variability among the studies.

The limit meta-analysis also included 22 studies with 881 observations. The adjusted estimate yielded an SMD of 0.05 (95% CI: −1.26 to 1.36, *z* = 0.08, *p*=0.937), indicating no significant effect. Substantial heterogeneity was noted (*τ*^2^ = 1.71, *I*^2^ = 91.3%, and *G*^2^ = 98.9%). The heterogeneity test was significant (*Q* = 184.77, df = 16, *p* < 0.001). The test of small-study effects was significant (*Q*-*Q*′ = 65.33, df = 1, *p* < 0.0001), indicating the presence of publication bias. Residual heterogeneity beyond small-study effects remained significant (*Q*′ = 119.43, df = 15, *p* < 0.001), highlighting substantial variability not explained by small-study effects.

Finally, a meta-regression analysis was performed to examine the effect of different moderators on the device intervention for NP. Our analyses revealed that the type of control comparator (whether sham or usual care; *R*^2^ = 0.143, *p* = 0.270) and funding type (*R*^2^ = 0.471, *p* = 0.320) did not exert a significant influence on the effect size. However, the etiology of NP (*R*^2^ = 0.588, *p* = 0.001) and the year of publication (*R*^2^ = 0.495, *p* = 0.009) were found to exert significant influence on the true effect size. The strength of synthesized evidence was found to be very low for both drug and device interventions based on the GRADE assessments (Tables 3 and 4).

#### 3.2.2. Relative Change From Baseline Intensity

**Figure 6:**
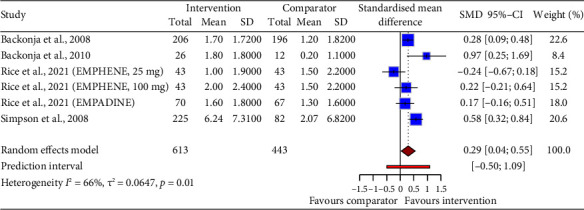
Meta-analysis of the change from baseline in neuropathic pain, comparing the effects of drug interventions to placebo. The figure shows the standardized mean differences (SMDs) and their 95% confidence intervals (CIs) for each study, as well as the overall effect size under a random-effects model. The zero line represents no effect of the intervention. The dotted line that extends to the red diamond shows the position of the overall effect size on the *x*-axis. The size of the blue squares reflects the study weight in the meta-analysis. The red diamonds show the pooled SMD for each drug and its width depicts the 95% CI. The red lines indicate the prediction intervals, which estimate the expected SMD and 95% CI for a new study to the meta-analysis.

We performed a meta-analysis of four studies that reported pain intensity as a change from baseline as an endpoint outcome measure (Figure 6). All six trials reported on drug-based interventions. The pooled estimate showed that drug interventions reduced pain intensity when considered as relative change from baseline in comparison to placebo interventions (SMD = 0.29, 95% CI: 0.04–0.55). The predictive interval ranged from −0.50 to 1.09, indicating a high degree of uncertainty and heterogeneity (*p*=0.01, *I*^2^ = 66%). The funnel plot showed significant asymmetry. Accordingly, the GRADE strength of evidence was found to be very low (Table 5).

### 3.3. Meta-Analysis of Secondary Outcomes

#### 3.3.1. Dizziness

**Figure 7:**
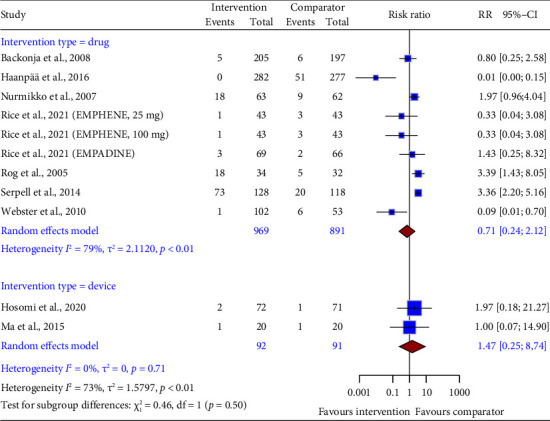
Forest plot of the risk ratio (RR) and 95% confidence interval (CI) for dizziness as an adverse event of drug and device interventions on neuropathic pain. The vertical line at RR = 1 represents no difference between the intervention and comparator groups. The size of the squares reflects the weight of each study in the meta-analysis. The horizontal lines represent the 95% CI for each study. The diamonds represent the pooled RR and 95% CI for each intervention type.

Pooled data from both drug (RR = 0.71, 95% CI: 0.24 to 2.12; Figure 7) and device interventions (RR = 1.47, 95% CI: 0.25–8.74) showed no difference in dizziness risk compared to control groups. Statistical heterogeneity was, however, noted only for drug intervention trials.

#### 3.3.2. Headache

**Figure 8:**
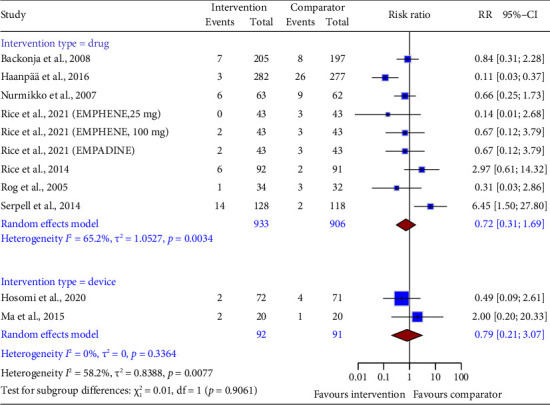
Forest plot of the risk ratio (RR) and 95% confidence interval (CI) for headache as an adverse event of drug and device interventions on neuropathic pain. The vertical line at RR = 1 represents no difference between the intervention and comparator groups. The size of the squares reflects the weight of each study in the meta-analysis. The horizontal lines represent the 95% CI for each study. The diamonds represent the pooled RR and 95% CI for each intervention type.

Pooled data from both drug (RR = 0.72, 95% CI: 0.31 to 1.69; Figure 8) and device interventions (RR = 0.79, 95% CI: 0.21–3.07) showed no difference in headache risk compared to control groups. Statistical heterogeneity was, however, noted for drug intervention trials.

#### 3.3.3. Hypertension

**Figure 9:**
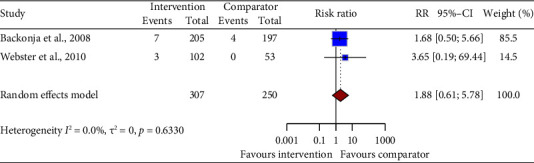
Forest plot of the risk ratio (RR) and 95% confidence interval (CI) for hypertension as an adverse event of drug interventions on neuropathic pain. The vertical line at RR = 1 represents no difference between the intervention and comparator groups. The size of the squares reflects the weight of each study in the meta-analysis. The horizontal lines represent the 95% CI for each study. The diamonds represent the pooled RR and 95% CI for each intervention type.

Pooled data from two drug intervention trials (RR = 1.88, 95% CI: 0.61 to 5.78; Figure 9) showed no difference in hypertension risk compared to control groups. Statistical heterogeneity was noted.

#### 3.3.4. Nausea

**Figure 10:**
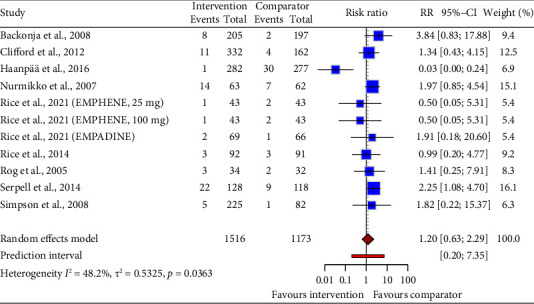
Forest plot of the risk ratio (RR) and 95% confidence interval (CI) for nausea as an adverse event of drug interventions on neuropathic pain. The vertical line at RR = 1 represents no difference between the intervention and comparator groups. The size of the squares reflects the weight of each study in the meta-analysis. The horizontal lines represent the 95% CI for each study. The diamonds represent the pooled RR and 95% CI for each intervention type.

Pooled data from 11 drug intervention trials (RR = 1.20, 95% CI: 0.63 to 2.29; Figure 10) showed no difference in nausea risk compared to control groups. Statistical heterogeneity, though significant, was within prespecified limits.

#### 3.3.5. Diarrhea

**Figure 11:**
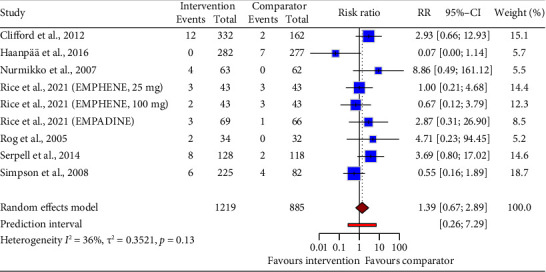
Forest plot of the risk ratio (RR) and 95% confidence interval (CI) for diarrhea as an adverse event of drug interventions on neuropathic pain. The vertical line at RR = 1 represents no difference between the intervention and comparator groups. The size of the squares reflects the weight of each study in the meta-analysis. The horizontal lines represent the 95% CI for each study. The diamonds represent the pooled RR and 95% CI for each intervention type.

Pooled data from nine drug intervention trials (RR = 1.39, 95% CI: 0.67 to 2.89; Figure 11) showed no difference in diarrhea risk compared to control groups. Statistical heterogeneity was not noted between the trials.

#### 3.3.6. Vomiting

**Figure 12:**
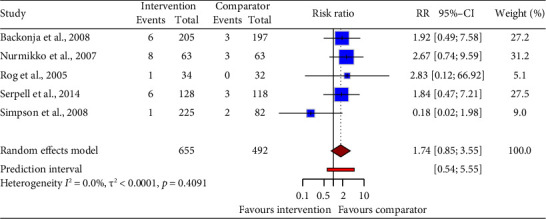
Forest plot of the risk ratio (RR) and 95% confidence interval (CI) for vomiting as an adverse event of drug interventions on neuropathic pain. The vertical line at RR = 1 represents no difference between the intervention and comparator groups. The size of the squares reflects the weight of each study in the meta-analysis. The horizontal lines represent the 95% CI for each study. The diamonds represent the pooled RR and 95% CI for each intervention type.

Pooled data from five drug intervention trials (RR = 1.74, 95% CI: 0.85 to 3.55; Figure 12) showed no difference in vomiting risk compared to control groups. Statistical heterogeneity was not noted between the trials.

## 4. Discussion

The main finding of this meta-analysis was that device interventions, especially rTMS, were more effective than sham, placebo, or usual care in reducing NP intensity. This finding is consistent with previous meta-analyses that have demonstrated the efficacy of rTMS for various types of NP [47]. As a noninvasive technique, rTMS modulates the activity of cortical neurons by applying magnetic pulses to the scalp [48, 49]. It is hypothesized that rTMS can alter the excitability and connectivity of the pain-related brain regions and induce neuroplastic changes that may restore the balance between inhibitory and facilitatory mechanisms [47, 50]. However, the optimal parameters of rTMS, such as the frequency, intensity, duration, and location of stimulation, remain to be determined by further research.

We also found no evidence of publication year bias in the effect size of device interventions, suggesting that their efficacy has been stable over time. However, this finding should be interpreted with caution, as the meta-regression may have been limited by the small number of studies and the narrow range of publication years. The source of heterogeneity may be attributed to the complex etiology and diversity of NP, time points, the differences in the device types and parameters, and the potential confounding factors such as medication use and psychological factors. Therefore, future studies should explore and investigate the potential moderators and mediators that may account for the variability in effect sizes across trials.

The findings from our meta-analysis of drug interventions demonstrate the inherent complexity of evaluating drug-based interventions for NP. The observed relative reduction in pain intensity with capsaicin 8% and EMA401, compared to placebo, suggests a potential benefit of these interventions when assessed using baseline measurements. However, significant heterogeneity and a broad predictive interval highlight the variability in patient responses and the uncertainty regarding the treatments' effectiveness. The very low GRADE strength of evidence further emphasizes the need for cautious interpretation.

The lack of notable discrepancies between the drug intervention and comparator groups regarding the absolute intensity of pain prompts questions regarding the clinical significance of the observed differences. This discrepancy between relative changes from baseline and absolute measures suggests that while some patients may experience pain relief, the overall impact on pain levels may not be substantial enough to differentiate drug effects from placebo in absolute terms. This distinction between absolute and relative changes in pain perception suggests that relative change from baseline metrics, which adjust for initial pain levels, provide a more dynamic and sensitive measure of treatment efficacy, capturing clinically meaningful improvements that absolute outcomes might overlook.

Additionally, it is pertinent to note that the differences in pain intensity outcomes for NP may arise from the specific drugs included in the analysis and the number of trials that were pooled. The meta-analysis assessing relative change from baseline included capsaicin 8% and EMA401, whereas the absolute outcome analysis involved Sativex and EMA401. Our findings highlight the importance of considering appropriate outcome measures in clinical trials. Drugs that have cumulative or time-dependent effects, such as capsaicin, are better assessed using relative changes from baseline. These drugs may show a gradual improvement over time, and capturing this dynamic change is crucial for understanding their true efficacy. It is known that with repeated or extended use of capsaicin, there is an initial stimulation that is followed by a prolonged reduction in pain sensitivity due to desensitization of pain-sensitive neurons [51, 52].

For chronic conditions where symptoms fluctuate or even enter remission over time, changes from baseline are often more informative. Medications for conditions like rheumatoid arthritis or NP may benefit from this approach, as it captures the long-term clinical impact. Conversely, for drugs that are expected to have immediate effects, absolute measures might be more appropriate, especially in cases where the intervention is short-term, and the primary goal is to achieve a specific outcome swiftly. For example, analgesics like ibuprofen or acetaminophen, which provide quick pain relief, can be effectively evaluated using absolute measures at specific time points. With the inclusion of more studies on these drugs in the future, subgroup analyses may provide clearer insights into their effects on NP. Nonetheless, our analysis highlights the importance of considering both relative and absolute measures when evaluating pain interventions' efficacy.

We also assessed the effects of device and drug interventions on secondary outcomes, such as dizziness, headache, hypertension, nausea, diarrhea, and vomiting. These outcomes are important to consider, as they reflect the quality of life and satisfaction outcomes of the patients. We found no statistical difference between device or drug interventions and comparators in terms of these secondary outcomes. This may indicate that both types of interventions are well-tolerated and safe for NP patients. However, it is important to acknowledge that the lack of statistical significance does not necessarily equate to the absence of effect. Clinical conclusions should be drawn with caution, as the studies may have been underpowered to detect small or rare adverse events or may have used inadequate or inconsistent methods to measure and report these outcomes.

Furthermore, the studies may have excluded patients with certain comorbidities or contraindications that could increase the risk of adverse events or may have used lower doses or shorter durations of the interventions that could reduce the occurrence of adverse events. Considering these limitations, the interpretation of secondary outcomes warrants careful consideration. The apparent nonsignificance in adverse event rates should not overshadow the potential clinical relevance of these outcomes. More comprehensive and rigorous assessment and reporting of secondary outcomes are needed in future trials to establish the true safety and tolerability profile of device and drug interventions for NP.

### 4.1. Limitations of the Study

This meta-analysis has some limitations that warrant consideration. A potential drawback is that we focused on pain intensity as the primary outcome measure, which may not reflect the full spectrum of NP and its consequences on quality of life, function, and psychological well-being. A previous study under the auspices of IMMPACT suggested that other relevant outcome measures, such as pain interference, emotional functioning, and patient satisfaction, may capture different aspects of chronic pain and its treatment effects [53]. Therefore, future meta-analyses should include these outcome measures to provide a more comprehensive and holistic assessment of NP and its interventions.

Moreover, we compared device or drug interventions with sham, placebo, or active comparators, which may not represent the real-world practice of using combination or sequential treatments for NP. For example, some studies have shown that combination therapy may have synergistic effects on pain [1, 54]. Future meta-analyses could examine the relative and combined effects of various types or modes of interventions on efficacy and safety outcomes, such as TENS, rTMS, and SCS, either alone or in combination with drugs. Another source of uncertainty is that we included only studies that were published in English, which may introduce language bias and exclude relevant studies from other regions or populations. A study from Zimmer et al. [55] reported that the prevalence and characteristics of pain may vary across different countries and cultures.

Future works should include studies that were published in other languages, or use multilingual databases and translators, to reduce the risk of language bias and increase the diversity and representativeness of the sample. A further challenge is that we included only studies that reported the effects of device or drug interventions at the end of the treatment period, which may not capture the long-term or sustained effects of the interventions. NP is a chronic condition that may require long-term management and follow-up. Subsequent systematic reviews should include studies that reported the effects of device or drug interventions at different time points, such as follow-up or maintenance periods, to examine the durability and stability of the treatment effects. On the other hand, we included studies that reported the effects of device or drug interventions on NP of any cause, which may account for the heterogeneity of NP mechanisms, phenotypes, and subtypes.

NP can result from various etiologies, such as diabetic neuropathy, postherpetic neuralgia, or spinal cord injury, which may have different pathophysiological and clinical features, and this may have accounted for the heterogeneity seen in our study. Later comprehensive syntheses should include studies that reported the effects of device or drug interventions on specific types or causes of NP, to identify the most suitable and effective interventions for each pain condition. Furthermore, given the noninvasive nature of the drug interventions and most device interventions, trials tended to report only quality of life outcomes as proxy measure for safety and tolerability of these interventions.

These limitations should be considered when interpreting and generalizing our findings. Despite these limitations, our meta-analysis provides a valuable and rigorous synthesis of the current evidence on the effects of device or drug interventions for NP. Our meta-analysis contributes to the advancement of knowledge and practice in the field of NP research and offers useful insights and directions for future studies and clinical applications.

### 4.2. Implications for Practice and Research

Our findings have important implications for the clinical practice and research of NP. For clinicians, our results suggest that device interventions, especially rTMS, might be considered as an alternative or adjunctive treatment option for patients with NP who do not respond to or tolerate conventional pharmacological therapies. However, the availability and accessibility of device interventions might be limited by the cost, expertise, and equipment required. Therefore, more studies are needed to evaluate the cost-effectiveness, feasibility, and safety of device interventions in different settings and populations. For researchers, our results highlight the need for more high-quality and standardized studies on the therapeutic modalities and pharmacotherapies for NP.

### 4.3. Future Research Directions

Our study has several implications for future research in the field of NP. We suggest the following recommendations:

• To establish the efficacy of device and drug interventions for NP, more high-quality RCTs are needed, following standardized protocols, outcome measures, and reporting guidelines.
• To optimize the delivery and effects of interventions for NP, further research should investigate the optimal dose or parameters, frequency, duration, and target of stimulation, using biomarkers and clinical predictors as indicators of response.
• To evaluate the value and feasibility of device and drug interventions for NP, comparative studies should assess the cost-effectiveness, feasibility, and acceptability of these interventions with pharmacological or nonpharmacological treatments, using patient-centered and health system perspectives.
• To develop and validate new treatments for NP, translational and personalized research should explore new therapeutic modalities and pharmacotherapies that target the specific mechanisms and pathways of NP.
• To identify the best candidates for device or drug interventions for NP, stratified and tailored research should examine the subgroups of patients who are most likely to benefit from these interventions, based on their pain phenotype and preferences.
• To measure the impact of device and drug interventions for NP, comprehensive and rigorous research should assess and report the effects of these interventions on secondary outcomes, such as quality of life, function, and psychological well-being.
• To determine the durability and generalizability of device and drug interventions for NP, consistent and relevant research should include and report the effects of these interventions at different time points, such as follow-up or maintenance periods.
• To specify the applicability and suitability of device and drug interventions for NP, clear and valid research should include and report the effects of these interventions on specific types or causes of NP, using clear and valid criteria and classifications.

## 5. Conclusions

Although drug interventions showed no effect in reducing the absolute pain intensity, when considered as relative change from baseline, drug interventions were found to be effective. This distinction is crucial as it highlights the importance of choosing appropriate outcome measures to capture the true efficacy of interventions, particularly for chronic conditions like NP. On the other hand, from device-based interventions, rTMS was found to be more effective than comparators in reducing absolute pain intensity. We believe that rTMS offers a promising alternative for managing NP, potentially improving clinical outcomes where other treatments fall short. However, we identified several methodological limitations and challenges in the current evidence base, such as heterogeneity, uncertainty, publication bias, lack of secondary outcomes, and poor outcome measurement. We recommend that future research should improve the quality and reporting of trials and use more comprehensive and patient-oriented outcome measures.

## Figures and Tables

**Table 1:** PICOS scheme employed in this review.

Component	Identifier
Patient	Individuals with neuropathic pain of any etiology or duration

Intervention	Device-based-rTMS, SCS, and TENS Drug-based-EMA401, Qutenza (capsaicin 8% patch), and Sativex

Control	Placebo, sham, usual care, or another standard active intervention on NP

Outcome (primary)	Pain intensity or change from baseline as measured by NRS, VAS, VRS, NPS, LANSS, FPS, MPQ, or any validated scale

Study design	Parallel RCTs

Abbreviations: FPS = Faces Pain Scale, LANSS = Leeds Assessment of Neuropathic Symptoms and Signs, MPQ = McGill Pain Questionnaire, NP = neuropathic pain, NPS = Neuropathic Pain Scale, NRS = Numeric Rating Scale, RCTs = randomized controlled trials, rTMS = repetitive transcranial magnetic stimulation, SCS = spinal cord stimulation, TENS = transcutaneous electrical nerve stimulation, VAS = Visual Analog Scale, and VRS = Verbal Rating Scale.

**Table 2:** Summary of the included randomized controlled trials in the present review.

Author, year	Trial name	Study design	Etiology of neuralgia	Primary endpoint	Time period	Location	Funding source	Trial registration no.
Cheing and Luk, 2005	TENS for neuropathic pain	Single-blind, parallel assignment randomized trial	Not reported	Effectiveness of high frequency TENS in reducing hypersensitivity of hands of people suffering from post-traumatic peripheral nerve injuries	Aug 2001 to May 2002	Hong Kong special administrative region	Not reported	Not reported

Rog et al., 2005	Randomized, controlled trial of cannabis-based medicine in central pain in multiple sclerosis	Double-blind, parallel assignment randomized trial	Multiple sclerosis	Change in pain intensity measured by comparing the mean score of the 7 days before the first dose (baseline) and the mean score of the last 7 days before the final intake of test medication	March 2002 to July 2002	United Kingdom	Private company	Not reported

Kumar et al., 2007	The prospective randomized controlled multicenter trial of the effectiveness of spinal cord stimulation (PROCESS)	Open-label, parallel assignment randomized trial	Failed back surgery syndrome	Proportion of patients achieving ≥ 50% leg pain relief at 6 months	April 2003 to June 2005	12 centers across Europe, Canada, Australia, and Israel	Private company	ISRCTN77527324

Nurmikko et al., 2007	Sativex successfully treats neuropathic pain characterized by allodynia	Double-blind, parallel assignment randomized trial	Allodynia	Change from baseline on NRS of mean intensity of global neuropathic pain	Not reported	6 centers across the UK and Belgium	Private company	Not reported

Backonja et al., 2008	Study of NGX-4010 for the treatment of postherpetic neuralgia	Phase-3, double-blind, parallel assignment randomized trial	Postherpetic	Percentage change in NRS score from baseline (measured 14 days before treatment) and the mean of the scores during weeks 2–8	June 2005 to Aug 2006	55 centers across the United States	Private company	NCT00115310

Simpson et al., 2008	Study of NGX-4010 for the treatment of painful HIV-associated neuropathy	Phase-3, double-blind, parallel assignment randomized trial	HIV-associated	Percent change in the average pain for the past 24 h NRS score from baseline to weeks 2–12	Aug 2003 to Dec 2005	30 centers across the United States	Private company	NCT00064623

Backonja et al., 2010	NGX-4010, a high-concentration capsaicin patch, for the treatment of postherpetic neuralgia	Double-blind, parallel assignment randomized trial followed by an open-label extension study	Postherpetic	For the double-blind trial—change from baseline (1–10 days before treatment) in the mean morning and evening NRS scores during days 8–28; for the extension study —(1) change in mean morning and evening NRS scores from baseline during weeks 2–12 after initial treatment; (2) change in mean morning and evening NRS scores from baseline to termination of the extension study; and (3) change in mean morning and evening NRS scores from baseline during weeks 2–12 for each treatment cycle in the extension study	Not reported	United States	Private company	Not reported

Webster et al., 2010	Controlled study of NGX-4010 for the treatment of postherpetic neuralgia	Phase-3, double-blind, parallel assignment randomized trial	Postherpetic	Percent change in average pain for the past 24 h measured using NRS scores from baseline to weeks 2 through 8	Not reported	19 centers across the United States	Private company	NCT00068081

Clifford et al., 2012	Study of NGX-4010 for the treatment of painful HIV-associated neuropathy	Phase-3, double-blind, parallel assignment randomized trial	HIV-associated distal sensory polyneuropathy	Percent change in NRS scores from baseline during weeks 2 through 12	Dec 2007	United States	Private company	NCT00321672

de Oliveira et al., 2014	rTMS of the left premotor/dorsolateral prefrontal cortex does not have analgesic effect on central poststroke pain	Double-blind, parallel assignment randomized trial	Central poststroke	Pain intensity measured using VAS at day 10	Not reported	Brazil	University	Not reported

Rice et al., 2014	A study of oral EMA401 in the treatment of pain following shingles to see if EMA401 can reduce the level of pain	Phase 2, double-blind, parallel assignment randomized trial	Postherpetic	Change in the mean pain intensity measured using NRS between baseline and the final week of dosing (days 22–28)	Aug 2011 to May 2012	29 centers across Bulgaria, Czechia, Georgia, Serbia, Ukraine, and South Africa	Private company	ACTRN12611000822987

Serpell et al., 2014	A study of Sativex® for pain relief of peripheral neuropathic pain, associated with allodynia	Phase-3, double-blind, parallel assignment randomized trial	Peripheral	Proportion of patients who achieve ≥ 30% decrease in pain measured using NRS from baseline to day 7	Sept 2005 to Oct 2006	39 centers across the UK, Czechia, Romania, Belgium, and Canada	Private company	NCT00710554

Yilmaz et al., 2014	The effect of rTMS on refractory neuropathic pain in spinal cord injury	Double-blind, parallel assignment randomized trial	Spinal cord injury	Change in pain intensity measured using VAS and satisfaction measured using Likert scale at baseline and up to 6 months after sessions	April 2010 to Jan 2012	Turkey	Not reported	Not reported

Bi et al., 2015	Effects of TENS on pain in patients with spinal cord injury	Parallel assignment randomized trial	Spinal cord injury	Change in pain intensity measuring using VAS score and MPQ before and 12 weeks after session	Not reported	China	Government	Not reported

Khedr et al., 2015	rTMS in neuropathic pain secondary to malignancy	Parallel assignment randomized trial	Secondary to malignancy	Pain relief (≥ 30% decrease) on the VAS after the 10^th^ session and 1 month later	Jan 2010 to May 2013	Egypt (multiple sites)	Author self-supported	Not reported

Ma et al., 2015	High-frequency rTMS reduces pain in postherpetic neuralgia	Parallel assignment randomized trial	Postherpetic	Change in pain intensity measured using VAS score and depression levels measured using SDS before, during, and up to 3 months after the sessions	Not reported	China	Not reported	Not reported

Haanpää et al., 2016	Capsaicin 8% patch versus oral pregabalin in patients with peripheral neuropathic pain (ELEVATE)	Double-blind, parallel assignment noninferiority randomized trial	Peripheral	Proportion of patients who achieve ≥ 30% decrease in average pain for the past 24 h measured using NRS from baseline to week 8	Not reported	92 sites across 22 countries in Europe and Asia	Private company	Not reported

Malavera et al., 2016	Effects of repetitive transcranial magnetic stimulation in the treatment of phantom limb pain in landmine victims (ANTARES)	Double-blind, parallel assignment randomized trial	Phantom limb pain	Change in pain score measured using VAS scores when compared with baseline and at 15 and 30 days after treatment including proportion of people with response (≥ 30% reduction in VAS scores)	Sept 2013 to Oct 2014	Colombia	Government	NCT01872481

Pei et al., 2019	rTMS at different frequencies for postherpetic neuralgia:	Double-blind, parallel assignment randomized trial	Postherpetic	Change in pain intensity measured using VAS score before, during, and up to 2 months after the sessions	Not reported	China	Government and university	Not reported

Sun et al., 2019	Analgesia-enhancing effects of rTMS on neuropathic pain after spinal cord injury	Double-blind, parallel assignment randomized trial	Spinal cord injury	Change in pain intensity measured using NRS and cortical oxyhemoglobin measured using functional near-infrared spectroscopy before and after the sessions	March 2018 to sept 2018	China	Government	Not reported

Hosomi et al., 2020	Analysis of efficacy and safety of rTMS of primary motor cortex in the intractable neuropathic pain	Double-blind, parallel assignment randomized trial	Not reported	Pain reduction measured by change in VAS score after every intervention session when compared with the baseline before the first intervention	Jan 2016 to Sept 2017	Japan (multiple sites)	Private company and government	UMIN000020291

Liu et al., 2020	Clinical study of spinal cord stimulation and pulsed radiofrequency for management of herpes zoster-related pain persisting beyond acute phase in elderly patients	Single-blind, parallel assignment randomized trial	Postherpetic	Change in pain intensity measured using NRS before operation and at multiple time points till 24 weeks post-operation. Proportion of participants with ≥ 50% change in NRS scores (effectiveness) and those with complete pain relief (NRS ≤ 3 points)	Jan 2018 to Jan 2019	China	Government	Not reported

Nabi et al., 2021	A comparison of the effectiveness of transcutaneous electrical nerve stimulation and duloxetine on diabetic peripheral neuropathic pain	Single-blind, parallel assignment randomized trial	Diabetic peripheral	Pain intensity change from baseline to 1 and 3 months post-treatment measured using NRS	Feb 2019 to April 2020	Iran	Author self-supported	IRCT20110413006186N13

Rice et al., 2021	Dose response study of EMA401 in patients with post-herpetic neuralgia (EMPHENE)	Phase-2b, quadruple-blind, parallel assignment randomized trial	Postherpetic	Change in weekly mean of the 24-h average pain score measured using NRS, from baseline to week 12	June 2017 to March 2019	45 sites across 19 countries	Private company	NCT03094195
	Safety and efficacy of EMA401 in patients with painful diabetic neuropathy (EMPADINE)	Phase-2b, triple-blind, parallel assignment randomized trial	Diabetic peripheral	Change in weekly mean of the 24-h average pain score measured using NRS, from baseline to week 12	March 2018 to March 2019	49 sites across 19 countries	Private company	NCT03297294

Viel et al., 2021	A study to compare Qutenza with pregabalin for the treatment of peripheral neuropathic pain (PNP) after 8 weeks of treatment (ELEVATE)	Phase-4, open-label, parallel assignment noninferiority randomized trial	Peripheral	Proportion of patients who achieve ≥ 30% decrease in average pain for the past 24 h measured using NRS from baseline to week 8	July 2011 to Sept 2013	92 sites across 22 countries in Europe and Asia	Private company	NCT01713426

Li et al., 2022	Prospective randomized controlled study on the efficacy of short-term spinal cord stimulation and pulsed radiofrequency for the treatment of postherpetic neuralgia	Single-blind, parallel assignment randomized trial	Postherpetic	Change in baseline pain intensity compared to postoperative pain intensity measured at multiple time points till 6 months	Sept 2019 to May 2020	China	University	ChiCTR2100050647

Sheng et al., 2022	Short-term spinal cord stimulation or pulsed radiofrequency for elderly patients with postherpetic neuralgia	Double-blind, parallel assignment randomized trial	Postherpetic	Proportion of patients with ≥ 50% reduction in VAS scores from baseline at the end of 360 days	Jan 2015 to Jan 2018	China	Not reported	Not reported

Yang et al., 2022	rTMS on diabetic peripheral neuropathic pain	Triple-blind, parallel assignment randomized trial	Diabetic peripheral	Pain intensity change from baseline to the end of 1-day and 1-week post-therapy using NRS	Not reported	South Korea	Government	NCT04833660

Kirmaci et al., 2023	Electrical stimulations on pain, functional capacity, and quality of life in multiple sclerosis	Single-blind, parallel assignment randomized trial	Multiple sclerosis	Pain intensity change from baseline to end of 4 weeks using VAS and LANSS questionnaire	Jan 2022 to Dec 2022	Turkiye	University	NCT05110586

Zubcevic et al., 2023	Tetra-hydro-cannabinol, cannabidiol and their combination for the treatment of peripheral neuropathic pain	Double-blind, parallel assignment randomized trial	Peripheral	Average daily pain intensity recorded every morning by NRS using a pain diary	Dec 2018 to May 2021	Denmark (multiple sites)	Government	EudraCT2017-005198-38

Abbreviations: ACTRN = Australian New Zealand Clinical Trials Registry, ChiCTR = Chinese Clinical Trial Registry, EudraCT = European Union Clinical Trials Register, IRCT = Iranian Registry of Clinical Trials, ISRCTN = International Standard Randomized Controlled Trial Number, LANSS = Leeds Assessment of Neuropathic Symptoms And Signs, MPQ = McGill Pain Questionnaire, NCT = National Clinical Trial Number as registered on United States Clinicaltrials.gov, NRS = Numeric Rating Scale, rTMS = repetitive transcranial magnetic stimulation, SDS = Self-Rating Depression Scale, TENS = transcutaneous electrical nerve stimulation, UMIN = University Hospital Medical Information Network Clinical Trials Registry (Japan), and VAS = Visual Analog Scale.

**Table 3:** GRADE summary of findings table for strength of evidence (pain intensity difference between device intervention and placebo).

Certainty assessment							No. of patients		Summary of findings		
No. of studies	Study design	Risk of bias	Inconsistency	Indirectness	Imprecision	Other considerations	Device	Comparator	Absolute (95% CI)	Certainty	Importance
17 trials reported in 16 studies	Randomized trials	Serious^1^	Serious^2^	Serious^3^	Not serious^4^	Publication bias suspected5	354	350	SMD 1.27 SD lower for device group (1.92 lower to 0.62 lower)	⨁◯◯◯ Very low∗	Critical6

^1^Rating was downgraded due to risk of bias arising from unclear or inadequate reporting of the randomization process in some studies. Refer to ROB2 analysis.

^2^Rating was downgraded due to high statistical heterogeneity, wide prediction interval, and some overlap of confidence intervals.

^3^Rating was downgraded due to differences in devices used, dosages, and etiology of neuropathic pain.

^4^According to GRADE handbook, for reviews reporting SMD as outcome, for considering a small effect size at *α* (0.05) and power (80%), the optimal information size (OIS) is approximately 200 participants per group (though cautious interpretation is suggested). Since we had > 200 participants in each group, a not serious rating was provided.

^5^Significant asymmetry was visualized in the funnel plot and using statistical tests as described in the results.

^6^Considered as critical based on PICOS scheme.

^∗^Interpretation: very low certainty indicates that we have very little confidence in the effect estimate, i.e., the true effect is likely to be substantially different from the estimate of effect.

**Table 4:** GRADE summary of findings table for strength of evidence (absolute pain intensity difference between drug intervention and placebo).

Certainty assessment							No. of patients		Summary of findings		
No. of studies	Study design	Risk of bias	Inconsistency	Indirectness	Imprecision	Other considerations	Drug	Comparator	Absolute (95% CI)	Certainty	Importance
3 trials reported in 3 studies	Randomized trials	Not serious	Serious^1^	Serious^2^	Serious^3^	Publication bias suspected4	188	185	SMD 1.21 SD lower for drug group (3.55 lower to 1.13 higher)	⨁◯◯◯ Very low∗	Critical5

^1^Rating was downgraded due to high statistical heterogeneity, wide prediction interval, and some overlap of confidence intervals.

^2^Rating was downgraded due to differences in drugs used, dosages, and etiology of neuropathic pain.

^3^According to GRADE handbook, for reviews reporting SMD as outcome, for considering a small effect size at *α* (0.05) and power (80%), the optimal information size (OIS) is approximately 200 participants per group (though cautious interpretation is suggested). Since we had < 200 participants in each group, a serious rating was provided.

^4^Although the number of studies was <10, a funnel plot was drawn to analyze publication bias visually. Significant asymmetry was visualized in the plot.

^5^Considered as critical based on PICOS scheme.

^∗^Interpretation: very low certainty indicates that we have very little confidence in the effect estimate, i.e., the true effect is likely to be substantially different from the estimate of effect.

**Table 5:** GRADE summary of findings table for strength of evidence (relative change in pain intensity from baseline levels).

Certainty assessment							No. of patients		Summary of findings		
No. of studies	Study design	Risk of bias	Inconsistency	Indirectness	Imprecision	Other considerations	Drug	Comparator	Absolute (95% CI)	Certainty	Importance
6 trials reported in 4 studies	Randomized trials	Not serious	Serious^1^	Serious^2^	Not serious^3^	Publication bias suspected4	613	443	SMD 0.29 SD higher for drug group (0.04 higher to 0.55 higher)	⨁◯◯◯ Very low∗	Critical5

^1^Rating was downgraded due to moderate statistical heterogeneity, wide prediction interval, and minimal overlap of confidence intervals.

^2^Rating was downgraded due to differences in drugs used, dosages, and etiology of neuropathic pain.

^3^According to GRADE handbook, for reviews reporting SMD as outcome, for considering a small effect size at *α* (0.05) and power (80%), the optimal information size (OIS) is approximately 200 participants per group (though cautious interpretation is suggested). Since we had > 200 participants in each group, a not serious rating was provided.

^4^Although the number of studies was < 10, a funnel plot was drawn to analyze publication bias visually. Significant asymmetry was visualized in the plot.

^5^Considered as critical based on PICOS scheme.

^∗^Interpretation: very low certainty indicates that we have very little confidence in the effect estimate, i.e., the true effect is likely to be substantially different from the estimate of effect.

## Data Availability

The data that support the findings of this study are available in the supporting information of this article.

## Supporting Information

Supporting Information 1 Description of any changes made and deviations to the original protocol and the reasons for them; search strategy; and a detailed account of the search terms, databases, and filters used to identify the relevant studies for the systematic review.

Supporting Information 2 Extracted data of the articles, including the study characteristics and outcomes.

Supporting Information 3 Compiled data used for the meta-analysis, including the study IDs, intervention groups, mean, and standard deviation.

Supporting Information 4 R code used for the meta-analysis and the generation of forest plots and funnel plots.

Supporting Information 5 PRISMA checklist with extended abstract checklist for systematic reviews and meta-analyses.
