# Supplementary material for: Emerging Therapeutic Modalities and Pharmacotherapies in Neuropathic Pain Management: A Systematic Review and Meta-Analysis of Parallel Randomized Controlled Trials
Source: Pain Res Manag. 2024 Dec 26;2024:6782574. doi: 10.1155/prm/6782574 (PMC11695085; doi:10.1155/prm/6782574)

**SUPPLEMENTARY MATERIAL**

**Emerging Therapeutic Modalities and Pharmacotherapies in Neuropathic Pain Management: A Systematic Review and Meta-analysis of Parallel Randomized Controlled Trials**

**SUPPLEMENTARY FILE 1**

- Deviations from the registered study protocol with justifications (Table 1)
- Search string and corresponding results per database

**Table 1.** Deviations from the registered study protocol with justifications

| Protocol method | Deviation from protocol method, with justification |
| --- | --- |
| Our initial title for the systematic review and meta-analysis was: Neurobiological Effects of Novel Therapies or Drugs Targeting Neuronal Excitability in Neuropathic Pain. | We revised the title to: Emerging Therapeutic Modalities and Pharmacotherapies in Neuropathic Pain Management: A Systematic Review and Meta-analysis of Randomized Controlled Trials.  The reason for this change was to make the title more concise, clear, and informative.  *Type of deviation: modification* |
| We planned to use Embase via Elsevier as one of the search databases for our systematic review. | Due to the unavailability of Embase via Elsevier for some of the reviewers, we used Embase via Ovid as a substitute. Embase via Ovid is a similar database that covers the same biomedical literature as Embase via Elsevier.  *Type of deviation: substitution* |
| Protocol method: We planned to include the following device and drug interventions in our systematic review, based on our initial literature search: Virtual reality (VR), Repetitive transcranial magnetic stimulation (rTMS), Deep brain stimulation (DBS), Spinal cord stimulation (SCS), Transcutaneous electrical nerve stimulation (TENS), Percutaneous electrical nerve stimulation (PENS), Epidiolex (cannabidiol), EMA401, TV-45070, Tiglutik,Qutenza (capsaicin 8% patch),Sativex | After conducting a pilot search, we decided to exclude the following interventions from our review, due to the lack of sufficient evidence (there should be at least 2) that was parallel randomized trials and met disease of interest: VR, DBS, PENS, Epidiolex, TV-45070, Tiglutik.  *Type of deviation: omission* |
| We intended to use both Revman and R software for the data analysis of our systematic review. | We decided to use only R software for the data analysis, as it could perform all the required statistical methods and produce reproducible code and results.  *Type of deviation: omission* |
| We planned to use odds ratio (OR) as the effect measure for dichotomous outcomes in our meta-analysis. | We decided to use risk ratio (RR) instead of OR for dichotomous outcomes, as it is easier to interpret and more intuitive for the reader [1].  *Type of deviation: substitution* |
| We planned to conduct sensitivity analysis to assess the robustness of our meta-analysis results and to explore the sources of heterogeneity. | We decided not to perform sensitivity analysis, as we performed subgroup analysis based on interventions. Sensitivity analysis would not have added any additional information or changed our conclusions in this case.  *Type of deviation: omission* |
| We did not plan to use meta-regression in our meta-analysis, as we did not anticipate any significant heterogeneity or confounding factors among the studies. | We decided to perform meta-regression of device intervention with year as a covariate, as we observed a high degree of heterogeneity (I^2 > 50%).  *Type of deviation: addition* |
|  |  |
| The protocol specified nine adverse events of interest: headaches, dizziness, hypertension, nausea, vomiting, insomnia, depression, death, and diarrhea. | Due to the limited availability of data on some of the adverse events, we decided to exclude insomnia, depression, and death from the analysis. The final set of adverse events included in the meta-analysis were dizziness, headache, hypertension, nausea, diarrhea, and vomiting.  *Type of deviation: omission* |

**Reference**

[1] J. P. Higgins and S. Green, "Cochrane handbook for systematic reviews of interventions," 2008.

**Search string with corresponding results per database**

**a. Pudmed Search Query: 304**

(neuropathic pain[Title/Abstract] OR neuropathic pain[MeSH Terms]) AND ((sativex[Title/Abstract] OR nabiximol[Title/Abstract] OR cannabinoid[Title/Abstract] OR tetrahydrocannabinol[Title/Abstract] OR GW-1000-02[Title/Abstract] OR cannabidiol[Title/Abstract] OR cannabinoid[MeSH Terms]) OR (capsaicin[Title/Abstract] OR "capsaicin 8%"[Title/Abstract] OR NGX-4010[Title/Abstract] OR Qutenza[Title/Abstract] OR capsaicin[MeSH Terms]) OR (rTMS[Title/Abstract] OR "repetitive transcranial magnetic stimulation"[Title/Abstract]) OR (TENS[Title/Abstract] OR "transcutaneous electrical nerve stimulation"[Title/Abstract]) OR (spinal cord stimulation[MeSH Terms] OR spinal cord stimulation[Title/Abstract])OR(olodanrigan[Title/Abstract] OR EMA401[Title/Abstract])) AND ("clinical trial"[Publication Type] OR "randomized controlled trial"[Publication Type]) AND ("english"[Language])

**b. Ebsco (CINAHL): 205**

((TI "neuropathic pain" OR AB "neuropathic pain") OR (MH "neuropathic pain+")) AND (((TI sativex OR AB sativex) OR (TI nabiximol OR AB nabiximol) OR (TI cannabinoid OR AB cannabinoid) OR (TI tetrahydrocannabinol OR AB tetrahydrocannabinol) OR (TI GW-1000-02 OR AB GW-1000-02) OR (TI cannabidiol OR AB cannabidiol) OR (MH cannabinoid+)) OR ((TI capsaicin OR AB capsaicin) OR (TI "capsaicin 8%" OR AB "capsaicin 8%") OR (TI NGX-4010 OR AB NGX-4010) OR (TI Qutenza OR AB Qutenza) OR (MH capsaicin+)) OR ((TI rTMS OR AB rTMS) OR (TI "repetitive transcranial magnetic stimulation" OR AB "repetitive transcranial magnetic stimulation")) OR ((TI TENS OR AB TENS) OR (TI "transcutaneous electrical nerve stimulation" OR AB "transcutaneous electrical nerve stimulation")) OR ((MH "spinal cord stimulation") OR (TI "spinal cord stimulation" OR AB "spinal cord stimulation")) OR ((TI olodanrigan OR AB olodanrigan) OR (TI EMA401 OR AB EMA401))) AND ((PT "clinical trial") OR (PT "randomized controlled trial")) AND ((LA english))

**c. Web of Science Core collection: 485**

((TI="neuropathic pain" OR AB="neuropathic pain") OR ALL="neuropathic pain") AND (((TI=sativex OR AB=sativex) OR (TI=nabiximol OR AB=nabiximol) OR (TI=cannabinoid OR AB=cannabinoid) OR (TI=tetrahydrocannabinol OR AB=tetrahydrocannabinol) OR (TI=GW-1000-02 OR AB=GW-1000-02) OR (TI=cannabidiol OR AB=cannabidiol) OR ALL=cannabinoid) OR ((TI=capsaicin OR AB=capsaicin) OR (TI="capsaicin 8%" OR AB="capsaicin 8%") OR (TI=NGX-4010 OR AB=NGX-4010) OR (TI=Qutenza OR AB=Qutenza) OR ALL=capsaicin) OR ((TI=rTMS OR AB=rTMS) OR (TI="repetitive transcranial magnetic stimulation" OR AB="repetitive transcranial magnetic stimulation")) OR ((TI=TENS OR AB=TENS) OR (TI="transcutaneous electrical nerve stimulation" OR AB="transcutaneous electrical nerve stimulation")) OR ((ALL="spinal cord stimulation") OR (TI="spinal cord stimulation" OR AB="spinal cord stimulation")) OR ((TI=olodanrigan OR AB=olodanrigan) OR (TI=EMA401 OR AB=EMA401))) AND (ALL="clinical trial" OR ALL="randomized controlled trial") AND (ALL=english)

**d. Medline via Ovid: 287**

("neuropathic pain".tw. OR exp "neuropathic pain"/) AND ((sativex.tw. OR nabiximol.tw. OR cannabinoid.tw. OR tetrahydrocannabinol.tw. OR GW-1000-02.tw. OR cannabidiol.tw. OR exp cannabinoid/) OR (capsaicin.tw. OR "capsaicin 8%".tw. OR NGX-4010.tw. OR Qutenza.tw. OR exp capsaicin/) OR (rTMS.tw. OR "repetitive transcranial magnetic stimulation".tw.) OR (TENS.tw. OR "transcutaneous electrical nerve stimulation".tw.) OR (exp "spinal cord stimulation"/ OR "spinal cord stimulation".tw.) OR (olodanrigan.tw. OR EMA401.tw.)) AND ("clinical trial".pt. OR "randomized controlled trial".pt.) AND (english.lg.)

**e. Scopus: 444**

( TITLE-ABS ( "neuropathic pain" ) OR INDEXTERMS ( "neuropathic pain" ) ) AND ( ( TITLE-ABS ( sativex ) OR TITLE-ABS ( cannabinoid ) OR TITLE-ABS ( tetrahydrocannabinol ) OR TITLE-ABS ( gw-1000-02 ) OR TITLE-ABS ( cannabidiol ) OR INDEXTERMS ( cannabinoid ) ) OR ( TITLE-ABS ( capsaicin ) OR TITLE-ABS ( "capsaicin 8%" ) OR TITLE-ABS ( ngx-4010 ) OR INDEXTERMS ( capsaicin ) ) OR ( TITLE-ABS ( "repetitive transcranial magnetic stimulation" ) ) OR ( TITLE-ABS ( tens ) OR TITLE-ABS ( "transcutaneous electrical nerve stimulation" ) ) OR ( INDEXTERMS ( "spinal cord stimulation" ) OR TITLE-ABS ( "spinal cord stimulation" ) ) OR ( TITLE-ABS ( ema-401 ) ) ) AND ( INDEXTERMS ( "clinical trials" OR "clinical trials as a topic" OR "randomized controlled trial" OR "Randomized Controlled Trials as Topic" OR "controlled clinical trial" OR "Controlled Clinical Trials" OR "random allocation" ) OR TITLE-ABS ( clinical AND trial* OR trial* OR rct* OR random* ) ) AND ( LIMIT-TO ( DOCTYPE , "ar" ) ) AND ( LIMIT-TO ( LANGUAGE , "English" ) ) AND ( LIMIT-TO ( EXACTKEYWORD , "Humans" ) OR EXCLUDE ( EXACTKEYWORD , "Animals" ) )

**f. Embase via Ovid: 278**


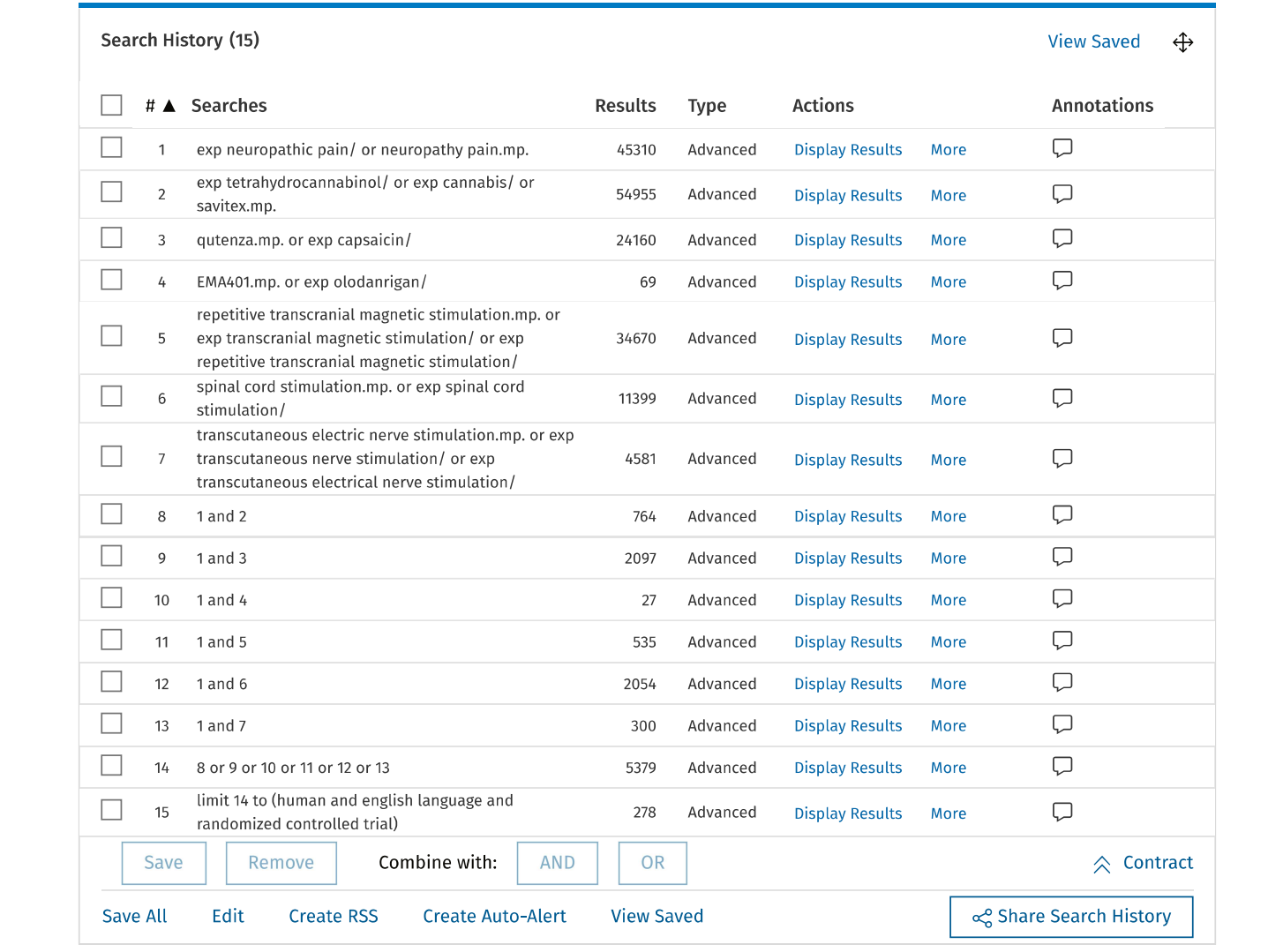

Supplement: Supporting Information 1 — Description of any changes made and deviations to the original protocol and the reasons for them; search strategy; and a detailed account of the search terms, databases, and filters used to identify the relevant studies for the systematic review. [file 6782574.f1.docx]
